# Respiratory‐ and cardiac motion‐corrected simultaneous whole‐heart PET and dual phase coronary MR angiography

**DOI:** 10.1002/mrm.27517

**Published:** 2018-10-15

**Authors:** Camila Munoz, Radhouene Neji, Karl P. Kunze, Stephan G. Nekolla, Rene M. Botnar, Claudia Prieto

**Affiliations:** ^1^ King’s College London, School of Biomedical Engineering and Imaging Sciences London United Kingdom; ^2^ Siemens Healthcare, MR Research Collaborations Frimley United Kingdom; ^3^ Technische Universität München, Nuklearmedizinische Klinik und Poliklinik Munich Germany; ^4^ DZHK (Deutsches Zentrum für Herz‐Kreislauf‐Forschung e.V.), partner site Munich Heart Alliance Munich Germany; ^5^ Pontificia Universidad Catolica de Chile, Escuela de Ingenieria Santiago Chile

**Keywords:** cardiac PET‐MR, dual‐phase coronary MRA, myocardial PET, respiratory and cardiac motion correction

## Abstract

**Purpose:**

To develop a framework for efficient and simultaneous acquisition of motion‐compensated whole‐heart coronary MR angiography (CMRA) and left ventricular function by MR and myocardial integrity by PET on a 3T PET‐MR system.

**Methods:**

An acquisition scheme based on a dual‐phase CMRA sequence acquired simultaneously with cardiac PET data has been developed. The framework is integrated with a motion‐corrected image reconstruction approach, so that non‐rigid respiratory and cardiac deformation fields estimated from MR images are used to correct both the CMRA (respiratory motion correction for each cardiac phase) and the PET data (respiratory and cardiac motion correction). The proposed approach was tested in a cohort of 8 healthy subjects and 6 patients with coronary artery disease. Left ventricular (LV) function estimated from motion‐corrected dual‐phase CMRA was compared to the gold standard estimated from a stack of 2D CINE images for the healthy subjects. Relative increase of signal in motion‐corrected PET images compared to uncorrected images was computed for standard 17‐segment polar maps for each patient.

**Results:**

Motion‐corrected dual‐phase CMRA images allow for visualization of the coronary arteries in both systole and diastole for all healthy subjects and cardiac patients. LV functional indices from healthy subjects result in good agreement with the reference method, underestimating stroke volume by 3.07 ± 3.26 mL and ejection fraction by 0.30 ± 1.01%. Motion correction improved delineation of the myocardium in PET images, resulting in an increased ^18^F‐FDG signal of up to 28% in basal segments of the myocardial wall compared to uncorrected images.

**Conclusion:**

The proposed motion‐corrected dual‐phase CMRA and cardiac PET produces co‐registered good quality images in both modalities in a single efficient examination of ~13 min.

## 1 INTRODUCTION

Since the development of simultaneous PET‐MR scanners, cardiovascular imaging has been proposed as one of the clinical applications that could greatly benefit from this new technology.^1^, ^2^, ^3^, ^4^, ^5^, ^6^ The high spatial and temporal resolution offered by MR and its superior soft‐tissue contrast, combined with the absolute quantification provided by PET has shown potential for improved diagnosis of different cardiac conditions, including coronary artery disease (CAD),^7^ cardiac sarcoidosis,^8^, ^9^, ^10^ and myocarditis.^11^, ^12^ Furthermore, some early reports^2^, ^13^ suggested that by simultaneously acquiring complementary information with both modalities, the cardiac PET‐MR clinical protocol could be optimized to reduce total scan time. However, image quality degradation due to physiological motion of both PET and MR images has prevented the clinical translation and adoption of cardiac PET‐MR yet.

Previously proposed motion compensation techniques for cardiac PET‐MR imaging have focused on improving PET image quality using motion information estimated from MR.^14^, ^15^ Several simulation, phantom, and patient studies have shown improved accuracy of PET uptake values in lesions and regions of interest and improved sharpness of anatomic structures such as the left ventricular myocardium.^16^, ^17^ Most of these approaches assume that both cardiac‐ and respiratory‐induced motion of the heart are periodic, and therefore it is possible to sort the data into near motion‐free frames by combining data acquired at similar cardiac phases and respiratory positions from multiple breathing and cardiac cycles, respectively. Using deformation fields obtained from non‐rigid registration of simultaneously acquired MR images, a motion‐compensated PET image can be reconstructed. Alternatively, a shorter MR acquisition can be performed at the beginning of the scan so that deformation fields are used to create a motion model that can be applied to longer PET acquisitions. This approach has been recently demonstrated in oncology patients, resulting in an increased uptake in liver, lung, and pancreatic lesions.^14^, ^15^

The main drawback of such approaches is that the MR images acquired simultaneously with PET are designed for motion estimation purposes only, so they have limited or no diagnostic value. Therefore, clinically useful MR images need to be acquired after the simultaneous PET‐MR examination leading to long acquisition times and misaligned diagnostic PET and MR images. Techniques for estimating respiratory^18^ and cardiac^19^ motion from the PET data itself have been proposed in the literature; however, they rely on correspondence between PET uptake and anatomical structure.

Recently, a novel approach where the motion estimated from MR is used to simultaneously correct both PET and MR images has been introduced.^20^, ^21^ The method proposed in Munoz et al.^20^ corrects for respiratory motion in both modalities and enables the simultaneous acquisition of myocardial viability PET and coronary MR angiography (CMRA) images. The approach proposed in Kolbitsch et al.^21^ obtains respiratory and cardiac motion‐compensated PET and anatomic MR images; however, MR images have reduced contrast between blood and myocardium because of the absence of preparation pulses and limited spatial resolution for coronary visualization.

In this work, we propose a simultaneous dual‐phase CMRA and cardiac PET data acquisition that provides: (1) coronary anatomy visualization from CMRA acquisition, (2) left ventricular function from a dual‐phase anatomic whole‐heart MR acquisition, and (3) assessment of myocardial viability or inflammation from cardiac and respiratory motion‐corrected ^18^F‐fluorodeoxyglucose (^18^F‐FDG) PET data, in a single efficient examination. This is achieved by extending the method proposed in Munoz et al.^20^ to correct for both cardiac and respiratory induced non‐rigid motion of the heart. Respiratory motion is estimated from 2D MR image navigators (iNAVs),^22^ acquired at each heartbeat before both the systolic and diastolic CMRA phases, and from high‐resolution 3D CMRA images reconstructed at different respiratory positions (so called bins) to estimate non‐rigid respiratory deformation fields. Furthermore, estimation of non‐rigid cardiac motion fields is achieved by registration of the systolic and diastolic images obtained from the proposed dual‐phase CMRA acquisition. The MR‐derived deformation fields are used to correct both the CMRA (respiratory motion correction for each cardiac phase) and the simultaneously acquired cardiac PET data (respiratory and cardiac motion correction). This approach is highly efficient, because it uses all the acquired PET and dual‐phase CMRA data for image reconstruction, and it produces good quality images with both modalities within a short and predictable acquisition time.

The proposed motion‐corrected dual‐phase CMRA reconstruction was tested in 8 healthy subjects. CMRA images were reformatted to visualize the coronary and cardiac anatomy, and left ventricular function (systolic and diastolic volumes, ejection fraction, and stroke volume) derived from the proposed dual‐phase 3D CMRA sequence was compared against gold standard values obtained from a conventional stack of 2D CINE images. The proposed respiratory and cardiac PET‐MR motion correction framework was tested in 6 cardiac patients with coronary artery disease, using ^18^F‐FDG for assessing either myocardial viability or inflammation.

## 2 METHODS

### 2.1 Image acquisition

**Figure 1:**
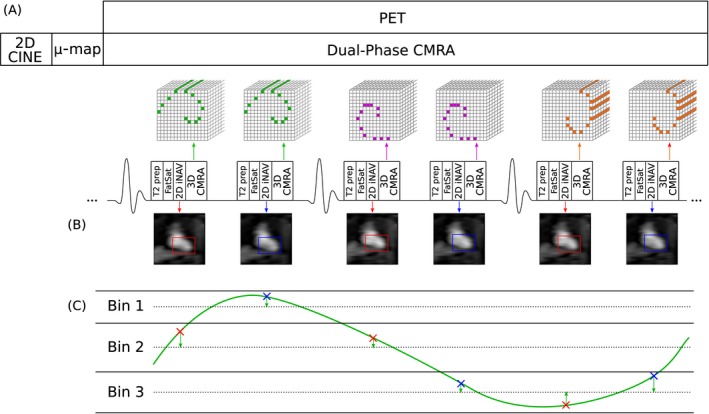
Simultaneous dual‐phase CMRA and cardiac PET acquisition scheme. (A) A 2D CINE image, used for defining the mid‐systolic and mid‐diastolic delays, and a MR‐based u‐map, used for attenuation correction of PET data, are obtained before the PET‐MR acquisition. Dual‐phase CMRA data is acquired following a golden‐step spiral ordering in a Cartesian grid, so that at each heartbeat, a spiral interleaf is acquired both in systole and diastole. Preparatory pulses (fat saturation [fat sat] and T_2_ preparation [T_2_ prep]) are used to enhance the contrast between the coronary arteries and surrounding tissues. (B) A low‐resolution coronal 2D image navigator (2D iNAV) is acquired before the 3D CMRA for estimation of foot‐head (FH) and right‐left (RL) respiratory motion for both cardiac phases (systole and diastole). (C) FH motion estimated from the iNAVs is used to define a number of respiratory bins, each containing the same amount of CMRA data, and FH and RL motion are used to correct the acquired CMRA data (2D translational correction) to the centre of each respiratory window

The proposed PET‐MR acquisition protocol consists of an electrocardiogram (ECG) triggered free‐breathing dual‐phase CMRA sequence simultaneously acquired with list‐mode PET data on a 3T hybrid PET‐MR system as shown in Figure 1A. Before the simultaneous PET‐MR acquisition, a standard Dixon‐based attenuation map (µ‐map) is acquired during breath‐hold at end‐expiration,^23^ and a 2D CINE acquisition is performed to determine two subject specific trigger delays that coincide with the mid‐systolic and mid‐diastolic quiescent periods of the cardiac cycle and the length of the acquisition window for 3D CMRA.

The dual‐phase CMRA data is acquired using a 3D spoiled gradient echo sequence with a fully sampled golden‐step Cartesian spiral profile order sampling trajectory,^24^ with 1 spiral interleaf acquired both in mid‐systole and mid‐diastole at each cardiac cycle, respectively. A low‐resolution 2D iNAV is acquired by adding spatially encoded low flip‐angle lines before each 3D CMRA acquisition. Preparatory pulses are performed before data acquisition to enhance the contrast between the coronary arteries and surrounding tissues: an adiabatic T_2_ preparation pulse is used to improve contrast between blood and myocardium without the use of exogenous contrast agents,^25^ and fat saturation is used to reduce the signal from subcutaneous and epicardial fat.

### 2.2 MR‐based respiratory and cardiac motion estimation and motion‐corrected dual‐phase CMRA reconstruction

Before PET and MR image reconstruction, the acquired 2D iNAVs are used to estimate the translational respiratory motion of the heart in the foot‐head (FH) and right‐left (RL) directions by tracking a rectangular template positioned around the apex of the heart (Figure 1B). The FH motion is then used to define ${\text{N}}_{\text{bins}}$ respiratory bins, ranging from end‐expiration to end‐inspiration, each containing approximately the same amount of dual‐phase CMRA data. CMRA data acquired during deep breaths are excluded from image reconstruction at this point, by rejecting data acquired outside 2 SDs from the mean FH translation.^20^, ^26^ Additionally, FH and RL translational motion estimates are used to pre‐correct the dual‐phase CMRA data to the centre of each respiratory bin by applying a linear phase to the k‐space data, as represented in Figure 1C (green arrows).

**Figure 2:**
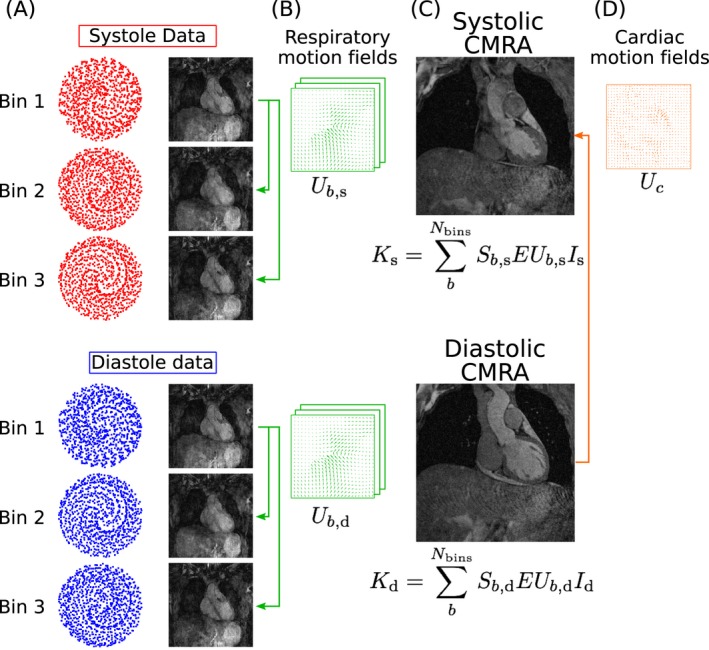
Motion‐corrected dual‐phase CMRA reconstruction scheme. (A) Respiratory bins (Figure 1C) are used to bin the dual‐phase CMRA. (B) MR images reconstructed at each cardiac and respiratory phase are used for estimation of non‐rigid respiratory motion fields (in green). (C) Respiratory motion‐corrected CMRA images for systole and diastole are reconstructed by including the respiratory motion fields in a generalized matrix description framework and used to estimate cardiac motion (D, in orange)

The motion estimation and respiratory motion‐corrected dual‐phase CMRA image reconstruction approach is shown in Figure 2. The pre‐corrected dual‐phase CMRA data is divided into the 2 cardiac phases (Figure 2A), and the following steps of the image reconstruction process^20^ are performed in each one of them separately. In the first step, the ${\text{N}}_{\text{bins}}$ undersampled bins are reconstructed with a soft‐binning iterative SENSE^27^ approach with exponential decay weighting by solving Equation (1) for each of them

(1) $${\hat{I}}_{b}={\text{argmin}}_{I_{b}}\left\{{W_{b}||EI_{b}-K_{b}\left|\right|}_{2}^{2}\right\},$$

where ${\hat{I}}_{b}$ are the reconstructed bin images, $W_{b}$ is a diagonal matrix containing data weights for bin *b*, E is the encoding operator, including the discrete Fourier transform and coil sensitivities, and $K_{b}$ is the data acquired at each respiratory bin after 2D translational motion correction to the center of the bin. The diagonal elements of $W_{b}$ are defined as an exponential decay function of the respiratory position where the k‐space data was acquired, so that points that belong to the respiratory bin being reconstructed will have a unitary weight, and points acquired outside the bin have a weight that decreases exponentially to zero as the distance to the center of the bin increases.

After the ${\text{N}}_{\text{bins}}$ respiratory bins have been reconstructed, 3D non‐rigid deformation fields that represent the respiratory motion are estimated in the second step (Figure 2B) via free‐form image registration using the end expiratory bin as reference, with normalized mutual information as similarity metric.^28^ Finally, the motion compensated image reconstruction problem is formulated using a generalized matrix description approach,^29^ so that if $I_{c}$ is the motion‐free image to be reconstructed for cardiac phase c (c=systole,diastole), then the motion‐corrupted measured k‐space can be expressed as

(2) $$K_{c}=\sum_{b}^{N_{\mathrm{bins}}}S_{b,c}EU_{b,c}I_{c},$$

where $U_{b,c}$ are motion operators that transform the cardiac phase c at the reference position to any respiratory position *b*, and $S_{b,c}$ corresponds to the sampling matrix containing the k‐space points acquired at respiratory bin b and cardiac phase c. Each motion free image is then reconstructed by solving Equation (2) for $I_{c}$ using a linear conjugate gradient method.^30^

At the end of the CMRA reconstruction process, one systolic and one diastolic respiratory motion‐corrected CMRA images are obtained, as shown in Figure 2C. These images are used for visualization of the coronary anatomy and estimation of ventricular function. Furthermore, these images are used to estimate the non‐rigid motion of the heart $U_{c}$ between systole and diastole, as shown in Figure 2D.

### 2.3 Motion‐corrected PET reconstruction

**Figure 3:**
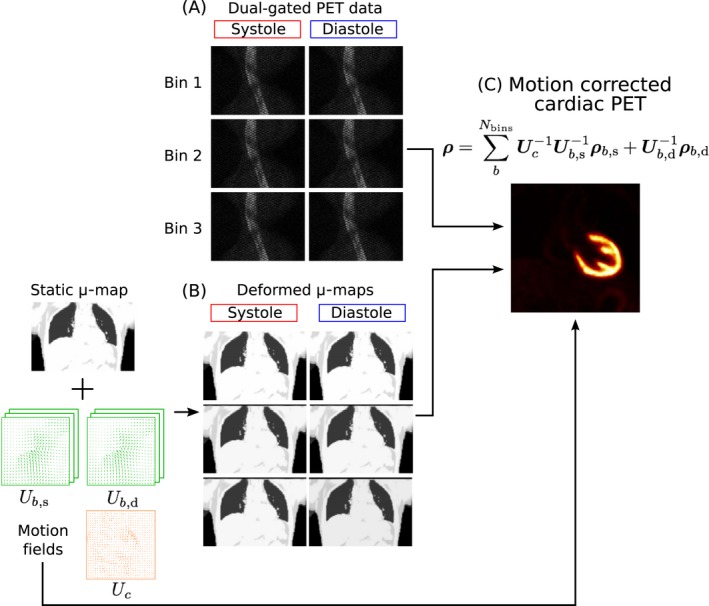
Motion‐corrected PET reconstruction scheme. (A) Respiratory bins (Figure 1C) and ECG signal are used to dual‐gate the PET data. (B) Respiratory and cardiac motion fields estimated from MR are used for transforming the static u‐map to each cardiac and respiratory position and (C) to produce a motion‐corrected PET image

List‐mode PET acquisition is synchronized with the dual‐phase CMRA acquisition using the ECG signal time stamps, so only PET data acquired simultaneously with MR is selected for image reconstruction. For each heartbeat, one‐third of the data acquired around maximum contraction of the left ventricle is assigned to the systolic phase, and the remaining two‐thirds are assigned to the diastolic phase. The cardiac binned PET data is then assigned to the corresponding respiratory bin, using the same respiratory windows defined in the dual‐phase CMRA reconstruction. Therefore, list‐mode PET data is dual‐gated into respiratory and cardiac phases as shown in Figure 3A.

The static attenuation map acquired at end‐expiration is transformed to each cardiac and respiratory phase using the corresponding cardiac and respiratory deformation fields estimated during CMRA reconstruction, as represented in Figure 3B. Using a post‐reconstruction‐registration (PRR) approach,^31^ each gate is independently reconstructed with an ordered‐subsets expectation‐maximization (OSEM) algorithm,^32^ including normalization, attenuation, random, and scatter corrections. Omitting the subsets division for simplicity, the iterative PET reconstruction algorithm can be written as

(3) $$\rho_{b,c}^{\left(it+1\right)}=\frac{\rho_{b,c}^{\left(it\right)}}{P^{T}A_{b,c}^{T}N^{T}1_{I}}P^{T}A_{b,c}^{T}N^{T}\frac{y_{b,c}}{NA_{b,c}P{\rho_{b,c}}^{\left(it\right)}+r+s},$$

where $\rho_{b,c}^{\left(it\right)}$ is a vector that contains the PET image for respiratory bin b and cardiac phase c ($b=1\cdots N_{\mathrm{bins}},\;c=\text{s, d}$) after *it* iterations of the algorithm, P is a matrix that models the system forward‐projection considering the scanner geometry, N and $A_{b,c}$ are diagonal matrices with entries down the diagonal equal to the reciprocal of the normalization and attenuation correction factors for respiratory bin b and cardiac phase c, respectively, $y_{b,c}$ is a vector that contains the dual‐gated data corresponding to respiratory bin b and cardiac phase c, and r and s represent estimations of random and scattered coincidences, respectively.

Finally, the images reconstructed at each respiratory and cardiac phase are transformed back to the diastolic and end‐expiratory phase (Figure 3C), and aggregated to produce a motion‐compensated PET image ρ, according to Equation (3). It is worth noting that only systolic images require transformation in the cardiac dimension, as the reference cardiac phase is diastole.

(4) $$\rho =\sum_{b=1}^{N_{\mathrm{bins}}}{U_{c}^{-1}U}_{b,\text{s}}^{-1}\rho_{b,\text{s}}+\;U_{b,\text{d}}^{-1}\rho_{b,\text{d}}.$$

### 2.4 Experiments

In vivo experiments were performed in 8 healthy subjects and 6 patients with known coronary artery disease on a 3T hybrid PET‐MR scanner (Biograph mMR, Siemens Healthcare, Erlangen, Germany). Written informed consent was obtained from all subjects according to institutional guidelines and the institutional ethics committee approved the study.

Eight healthy subjects (age 29.9 ± 3.4 y) were scanned during free breathing using a prototype implementation of the proposed dual‐phase CMRA sequence. The following acquisition parameters were used: coronal slices, RL phase encoding, 1 mm × 1 mm × 2 mm resolution, FOV 304 mm × 304 mm × 80–96 mm with subject‐specific number of slices covering the whole heart, TR/TE = 3.7/1.7 ms, flip angle = 12°, readout bandwidth = 685 Hz/px. Two subject‐specific trigger delays were set targeting the mid‐systolic and mid‐diastolic rest period and an acquisition window ranging from 82–104 ms (corresponding to 22–28 k‐space lines acquired per heartbeat) was used depending on the length of the quiescent periods of the subject, adiabatic T_2_ preparation pulse of 50 ms and fat saturation. For the 2D iNAV acquisition, the following parameters were used: high‐low Cartesian trajectory, coronal orientation, RL phase encoding, flip angle = 3°, 14 readouts with the same FOV of the CMRA acquisition, resulting in a 1 mm × 21.7 mm acquired in‐plane resolution (reconstructed to 1 mm × 1 mm).

Additionally, a conventional multi‐slice and multi‐breath‐hold 2D short‐axis CINE acquisition was performed and used as reference standard for left ventricular function estimation. The stack of 2D CINE images was acquired with an in‐plane resolution of 1.5 × 1.5 mm^2^, using GRAPPA parallel imaging with an undersampling factor of 2 and 44 calibration lines, slice thickness of 8 mm, 8–10 slices covering the whole left‐ventricle, and 25 cardiac phases.

Six patients with symptomatic CAD (angina or angina equivalent, excluding acute ST‐elevation myocardial infarction patients) and known chronic total occlusion of at least one of the coronary arteries (ages 66.3 ± 8.6 y) received an FDG injection of 330.7 ± 30.3 MBq and were scanned 1.73 ± 0.77 h after injection. The simultaneous PET‐CMRA acquisition was performed ~1–2 min after injection of a gadolinium‐based contrast agent during the 10–15 min waiting time required for optimal contrast in conventional late gadolinium enhancement images. Before the dual‐phase CMRA acquisition, a µ‐map was acquired during a 19‐s breath‐hold at end‐expiration using the vendor’s standard Dixon acquisition (imaging parameters: coronal orientation, FH phase encoding, TR/TE1/TE2 = 3.60/1.23/2.46 ms, 328 × 500 × 399 mm^3^ field of view, 2.604 × 2.604 × 3.12 mm^3^ resolution). Following this, the subjects were scanned during free breathing using the proposed dual‐phase CMRA sequence with the same acquisition parameters described for the healthy subjects. List‐mode PET data was acquired during the whole dual‐phase CMRA scan.

For all data sets, dual‐phase CMRA image reconstruction was performed offline in MATLAB (The MathWorks, Natick, MA) using custom‐developed software. CMRA data were reconstructed offline with the proposed motion correction approach (MC) and without motion correction (NMC) for comparison purposes. Image registration for estimating respiratory and cardiac motion operators was performed with the software package NiftyReg^28^ that provides both forward and inverse deformation fields. The MC dual‐phase CMRA reconstruction required 2D translational motion‐corrected soft‐binning iterative SENSE reconstructions to produce 3D images at different cardiac and respiratory phases (reconstruction time of ~3.6 min per bin), followed by 3D respiratory bin‐to‐bin non‐rigid registration at each cardiac phase (registration time of ~40 s per bin) and finally the motion‐corrected CMRA reconstruction (reconstruction time of ~30 min per cardiac phase), with a total reconstruction time of 103 min. Reconstruction of the stack of 2D CINE images was performed directly on the scanner.

PET image reconstruction was performed offline using MATLAB and e7 Tools (Siemens Healthcare, Knoxville, TN) using the OSEM algorithm, with 3 iterations and 21 subsets, point spread function modeling, voxel size = 2.03 × 2.08 × 2.08 mm^3^, matrix size = 127 × 344 × 344. PET images were reconstructed using both cardiac and respiratory motion correction (MC), using only respiratory motion correction (RespMC) and without motion correction (NMC) for comparison purposes. The MC PET reconstruction required OSEM reconstructions at each cardiac and respiratory phase (~4 min per bin), followed by 3D non‐rigid deformation (~10 s per bin) with a total reconstruction time of 42 min.

### 2.5 Image analysis

For assessment of image quality, reconstructed systolic and diastolic CMRA images were reformatted to visualize the left anterior descending (LAD) and right coronary artery (RCA) simultaneously, using dedicated software.^33^ For the healthy subjects, left ventricular function was estimated using dedicated commercially available software (OsiriX, Geneva, Switzerland). For the diastolic and systolic CMRA images, end‐diastolic (EDV) and end‐systolic (ESV) volumes were obtained from semi‐automatic segmentations of the left ventricular blood pool. For the 2D CINE images, EDV and ESV were obtained by semi‐automatically contouring the left ventricle. In both cases, stroke volume (SV) and ejection fraction (EF) were then derived by subtracting the ESV from the EDV and by taking the ratio between SV and EDV, respectively. A Bland‐Altman analysis was performed for each of the indices to assess the agreement between both measurements.

Reconstructed ^18^F‐FDG PET images were analyzed by using the AHA 17‐segment model for the left ventricular myocardium^34^ using dedicated software (MunichHeart).^35^ The relative increase in ^18^F‐FDG signal of RespMC and MC images over uncorrected images was computed for each of the 17 segments for each patient.

## 3 RESULTS

**Figure 4:**
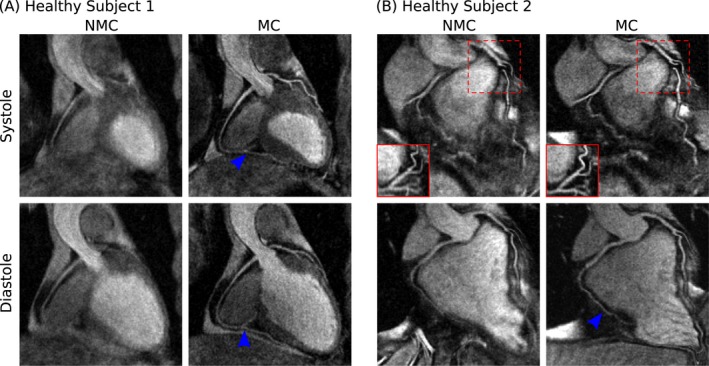
Reformatted dual‐phase CMRA images showing uncorrected (NMC) and motion‐corrected (MC) images for both systole and diastole in 2 representative healthy subjects. MC improves the visualization of both the left (LAD) and right coronary arteries (RCA), allowing for the depiction of both proximal and distal segments (blue arrows). In (B), the tortuous anatomy of the LAD in systole prevents the visualization of the artery in the reformatted coronal plane (dashed red box), but this is solved when reformatting in the transverse plane (solid red box)

**Figure 5:**
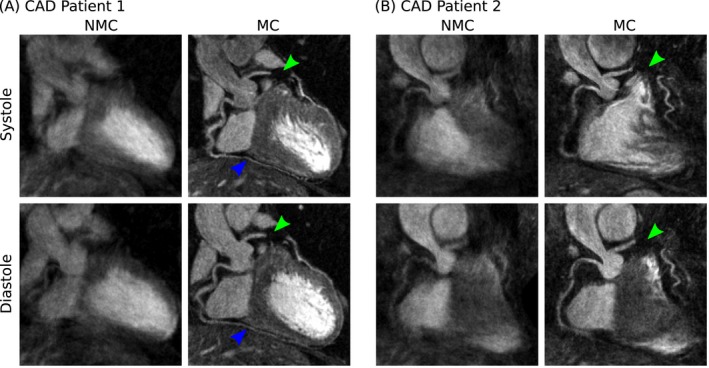
Reformatted dual‐phase CMRA images showing uncorrected (NMC) and motion‐corrected (MC) images for both systole and diastole in 2 representative patients with coronary artery disease. MC improves the visualization of the proximal left (LAD) and right coronary arteries (RCA) and in (A) allows for the depiction of non‐stented distal segments of the RCA (blue arrows). In both cases, the LAD can be clearly depicted despite the presence of a stent (green arrows) in the mid‐segment of the vessel

Scans were successfully completed in all subjects, with an average acquisition time of 12.72 ± 2.29 min for the proposed sequence. Figures 4 and 5 show reformatted uncorrected (NMC) and motion‐corrected (MC) systolic and diastolic CMRA images for representative healthy subjects and patients, respectively. It can be observed that MC improved the delineation of the vessels, allowing for visualization of both the RCA and LAD in all cases.

For the healthy subjects, it can be observed that MC increased the visible length of the vessels, allowing for visualization of the distal segment of the arteries in both systole and diastole (Figure 4A, blue arrows). For the second healthy subject, the tortuous anatomy of the LAD in systole prevented an appropriate reformatting in the coronal plane, as shown in Figure 4B (dashed red box). However, changing the reformatting plane solved this issue, and the vessel is clearly depicted in the transverse plane (Figure 4B, solid red box). In the CAD patients, who have more irregular breathing patterns and larger respiratory amplitude, improvements in the delineation of the vessels after MC became even more apparent. In both cases, the proximal RCA and LAD were well‐depicted in the MC images, and the presence of a stent in the mid segment of the LAD did not prevent visualization of the distal segment of the vessel (Figures 5A and 5B, green arrow). For the second CAD patient (Figure 5B), residual cardiac motion was observed in the MC systolic CMRA, affecting the sharpness of the mid and distal RCA.

**Figure 6:**
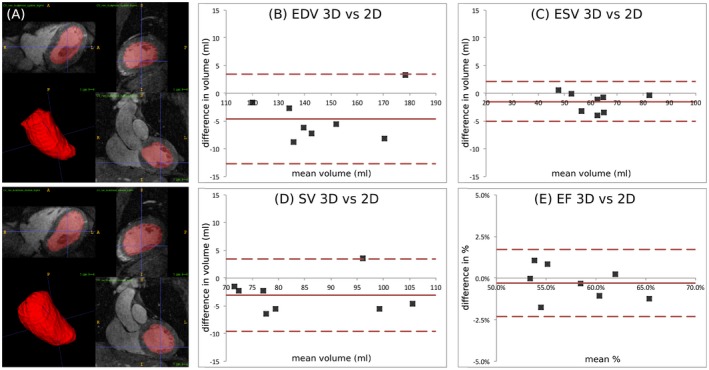
(A) Semi‐automatic segmentation for a representative subject, showing 3 orthogonal planes for systole and diastole and corresponding rendering of the left ventricular cavity. Bland‐Altman plots of (B) end diastolic volume (EDV), (C) end systolic volume (ESV), (D) stroke volume, (SV) and (E) ejection fraction (EF) comparing dual‐phase 3D CMRA images and conventional multi‐slice 2D CINE acquisition. Middle red line: mean difference; upper and lower dashed red lines: 95% limits of agreement

Conventional orthogonal planes obtained from MC systolic and diastolic CMRA images of a representative subject are shown in Figure 6A, in addition to the corresponding volumetric rendering of the left ventricle. It can be observed that the contrast between blood pool and myocardium was sufficient and allowed for segmentation of the left ventricular cavity in both cardiac phases. Figures 6B–6E shows Bland‐Altman plots for left ventricular volumes and indices obtained for the cohort of healthy subjects, indicating in each case the average bias (solid red line) and 95% limits of agreement (dashed red lines). EDV and ESV estimated from dual‐phase 3D CMRA images are comparable to the estimation from multi‐slice multi‐breath‐hold 2D cine images, with an average underestimation of 4.61 ± 4.03 mL and 1.54 ± 1.75 mL, respectively (Figures 6B and 6C). Functional indices obtained from dual‐phase CMRA therefore resulted in good agreement with the conventional 2D cine approach, with an average bias of −3.07 ± 3.26 mL and −0.30 ± 1.01% for the SV and EF, respectively (Figures 6D and 6E).

**Figure 7:**
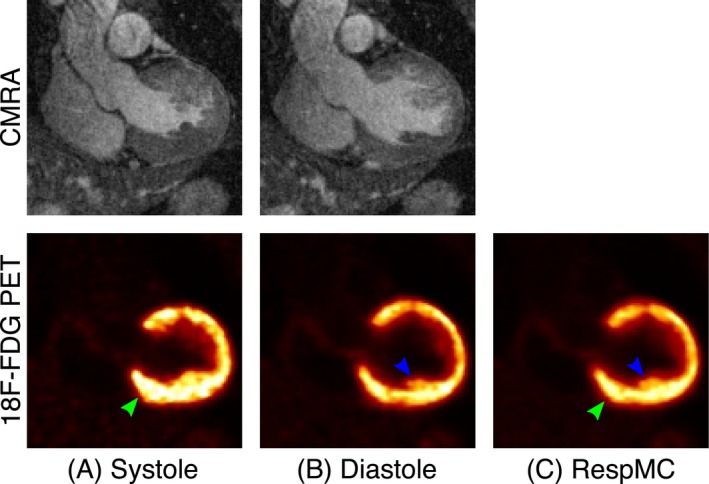
Coronal views from CAD patient 3 data set showing respiratory motion‐corrected (A) systolic and (B) diastolic CMRA and ^18^F‐FDG PET images alongside the (C) respiratory motion‐corrected (RespMC) PET image. RespMC aggregates data acquired at different cardiac phases, resulting in cardiac‐induced blurring of small structures (blue arrow) and apparent thickening of the myocardium (green arrow)

Respiratory motion‐corrected systolic and diastolic CMRA and PET images for a representative patient are shown in Figure 7, alongside the respiratory motion‐corrected (RespMC) PET image. Differences in the anatomy of the heart between the 2 cardiac phases are apparent in both imaging modalities, including thickening of the left ventricle myocardium in the systolic phase as compared to the diastolic phase. RespMC aggregates data acquired at both cardiac phases, resulting in cardiac‐induced blurring of small structures, such as the papillary muscles, compared to diastolic PET images (Figures 7B and 7C, blue arrow) and a thicker myocardium, because of signal arising from the systolic phase (Figures 7A–7C, green arrow). It is worth noting that the myocardium depicted by the RespMC image is dominated by the diastolic image, because diastole comprises a longer fraction of the cardiac cycle.

**Figure 8:**
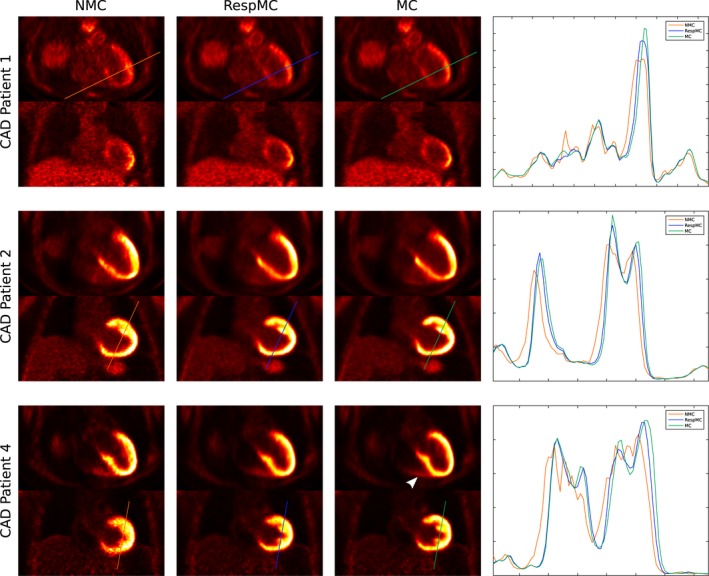
Uncorrected (NMC), respiratory motion‐corrected (RespMC), and cardiac and respiratory motion‐corrected (MC) cardiac PET images for 3 patients including transverse and coronal views alongside profiles across the myocardium. RespMC increases sharpness of the myocardium, and MC further improves sharpness, illustrating the impact of cardiac motion correction in both the left and right ventricle myocardium (white arrow)

Cardiac PET images reconstructed without motion‐correction (NMC), with respiratory motion correction only (RespMC), and with both cardiac and respiratory motion correction (MC) from 3 representative patients together with profiles across the left ventricle myocardium obtained from transverse slices are shown in Figure 8. RespMC improved image quality by increasing the sharpness of the myocardium in all cases, as can be observed in the narrowing of the peak in the profiles. MC further increased myocardial sharpness and improved delineation of features such as the papillary muscles (as can be observed for CAD patients 2 and 4) and the right ventricle myocardium (CAD patient 4, white arrow).

**Figure 9:**
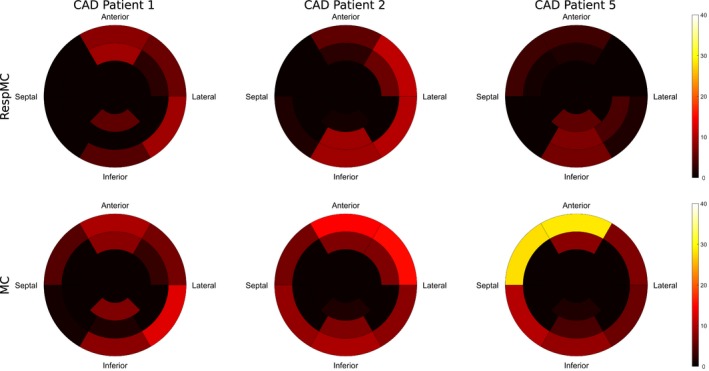
Seventeen‐segment polar maps of the relative increase in ^18^F‐FDG PET signal after respiratory motion correction only (RespMC) and after cardiac and respiratory motion correction (MC) for the left ventricular myocardium in 3 representative patients

Figure 9 shows 17‐segment plots for 3 representative patients, displaying the relative increase of the ^18^F‐FDG signal in each myocardial segment after RespMC and MC compared to uncorrected images. In all cases, an increased signal in the basal segments of the myocardial wall was observed after RespMC, with a maximum relative increase ranging from 6.8 to 11.2% compared to uncorrected images. Further increase was observed in MC images, ranging from 13.1 to 28.7%, the latter observed in the anteroseptal segments in CAD patient 5.

## 4 DISCUSSION

In this study, we have presented a novel approach for the simultaneous acquisition of coronary MR angiography, left ventricular function, and motion‐corrected cardiac PET. This approach extends the work presented in Munoz et al.^20^ to a dual‐phase acquisition, so that a systolic and a diastolic CMRA image are acquired in a single examination, allowing for quantification of left ventricular volumes and functional indices, in addition to providing cardiac motion deformation fields for motion correction of PET images. This approach is highly efficient, as it uses all the acquired data for image reconstruction (no data rejection) and produces co‐registered high quality images in both modalities from a single examination of ~12.7 min.

The proposed approach was tested in 8 healthy subjects (MR only acquisitions) and 6 patients with coronary artery disease (PET‐MR acquisition), and we observed improved delineation and increased visible length of both the RCA and LAD arteries after motion correction for all subjects. For the healthy subjects, proximal and distal segments of the coronary arteries are clearly depicted in both systole and diastole. It is worth noting the significant change in the coronary anatomy between the 2 cardiac phases: for one of the subjects (Figure 4B), a tortuous anatomy of the proximal LAD in systole prevented its visualization in the coronal view, requiring an additional view in the transverse plane for appropriate depiction of the vessel. For the CAD patients, improved delineation of the coronary arteries after motion correction was observed even for stented vessels, as shown in Figures 5A and 5B. In one of the patients, a short systolic quiescent period resulted in residual cardiac motion, as can be observed in Figure 5B. However, the coronary anatomy could be assessed in the diastolic CMRA, whereas the systolic CMRA still provided sufficient information for estimating cardiac motion.

Left ventricular function estimation was validated in the cohort of 8 healthy subjects, by comparing volume quantification and functional indices obtained from systolic and diastolic CMRA images to a conventional stack of 2D CINE images. Our results suggest that quantification of left ventricular function from dual‐phase CMRA data is in agreement with the reference method, with an average underestimation in stroke volume of 3.07 ± 3.26 mL and a 0.30 ± 1.01% underestimation of ejection fraction. These results are comparable to the study of Uribe et al.,^36^ where left ventricular volumes obtained from a prospectively EC‐triggered dual‐phase whole‐heart cardiac MR with a balanced steady‐state free‐precession acquisition performed on a 1.5T system were compared to conventional retrospectively gated 2D CINE images, finding an underestimation of 0.95 mL and 0.67% in stroke volume and ejection fraction, respectively. In this study, systolic and diastolic CMRA data sets were acquired during mid‐systole and mid‐diastole to minimize the effect of cardiac motion, however, this might produce an underestimation of the ventricular volumes compared with conventional CINE imaging. This bias could be reduced by acquiring data toward the end of systole and diastole.

Reconstructed cardiac PET images showed that motion correction improves image quality compared to uncorrected images. For all subjects, it was observed that respiratory motion correction increased the sharpness of the myocardium and definition of small features, and incremental improvements were obtained when both respiratory and cardiac motion correction was applied. This is consistent with findings in Küstner et al.,^15^ where minor visual improvements were observed after cardiac motion correction in patients with suspected liver or lung metastasis that exhibited myocardial uptake during the PET examination. Quantitative analysis of 17‐segment polar plots obtained from the reconstructed PET images showed that motion correction increased ^18^F‐FDG signal in the basal segments of the myocardial wall compared to uncorrected images. Across the whole cohort, maximum increase in the signal was observed when both cardiac and respiratory motion correction was applied, in the anterior and septal basal segments, corresponding to the regions of larger cardiac motion. This increase of signal corresponds to the combined effect of reduced image blurring and improved alignment between attenuation maps and emission data. As shown in Ouyang et al.^37^ for myocardial perfusion PET imaging, motion artefacts can appear as a myocardial defect, therefore in such cases the relative increase in PET signal coming from accurate motion correction allows for an accurate assessment of myocardial integrity.

In this study, the systolic and diastolic CMRA images were visually assessed in terms of coronary artery depiction before and after non‐rigid respiratory motion correction. The non‐rigid motion correction method has been previously validated against a conventional 1D diaphragmatic navigator gated and tracked acquisition on both a 1.5T MR system^26^ and a 3T PET‐MR system^20^ for vessel wall imaging and diastolic CMRA in healthy subjects, respectively, finding no statistically significant difference in visible vessel length and vessel sharpness between both acquisitions. Further validation of the respiratory motion‐corrected dual‐phase CMRA in a larger cohort of patients with known or suspected coronary artery disease will be performed in future studies, including a comparison between findings from our dual‐phase CMRA approach and a gold standard method such as invasive X‐ray angiography.

Conventionally, 3D CMRA imaging at 3T uses spoiled gradient‐echo sequences to minimize artefacts because of field inhomogeneity, relying on relatively long times between acquisitions (~800 ms for a heart rate of 60 bpm) to produce sufficient longitudinal magnetization recovery. For the dual‐phase CMRA approach, the time between acquisitions is reduced by 50% approximately compared to conventional diastolic CMRA, producing a penalty in SNR, especially in acquisitions without contrast agent injection. Acceleration techniques that reduce the length of the acquisition window without increasing total acquisition time would allow for increased longitudinal magnetization recovery between systolic and diastolic acquisition, thereby improving SNR of the images. Furthermore, such acceleration techniques could alleviate the problem of residual cardiac motion during systolic CMRA acquisition. Future work includes incorporating acceleration techniques and integrating arrhythmia rejection algorithms for further improving dual‐phase CMRA image quality.

The proposed framework provides visualization of the coronary anatomy in systole and diastole and ventricular function by dual‐phase CMRA and motion‐corrected myocardial viability by ^18^F‐FDG PET in a single examination. However, a comprehensive assessment of cardiac disease may require the acquisition of additional contrasts, for example LGE imaging for assessment of scar or T_2_ mapping for quantification of edema. In both cases, similar motion‐corrected approaches could be used, for example, by extending the approach proposed in Ginami et al.^38^ to a 3T PET/MR system, allowing for simultaneous visualization of the coronary anatomy and scar by MR and myocardial viability by ^18^F‐FDG PET. Similarly, simultaneous whole‐heart T2 mapping and myocardial viability by ^18^F‐FDG PET could be achieved by extending the approach proposed in Yang et al.^39^

Recently published approaches for cardiac and respiratory motion correction of PET data have proposed the use of 8–12^15^, ^21^ cardiac phases, whereas our approach considers motion correction only between the 2 extreme cardiac phases to ensure good coronary MR depiction in at least one of the cardiac phases. Preliminary results obtained from a simulation study^40^ suggest that accurate quantification of size and degree of transmurality of myocardial viability defects by PET can be obtained when correcting for cardiac motion using only the systolic and diastolic cardiac phases. However, for radiotracers targeted at different cardiac applications, such as ^18^F‐sodium fluoride for imaging of inflammation and calcification of coronary plaques, additional cardiac phases might be required for accurate motion correction of PET data and further investigation is needed.

The feasibility of the proposed framework was demonstrated in a small cohort of patients and further studies are now warranted to validate the whole‐heart PET‐dual‐phase CMRA method in larger cohorts of patients with heart disease.

## 5 CONCLUSION

A novel framework for the simultaneous acquisition of coronary MR angiography, left ventricular function, and motion‐corrected cardiac PET for hybrid whole‐heart PET‐MR imaging has been presented. Our approach produces co‐registered high quality images in both modalities in short and predictable scan time of ~12.7 min, and therefore is suitable for integration into clinical routine. Left ventricular function quantification has been validated in a cohort of healthy subjects, finding a good agreement between left ventricular volumes and functional indices from the dual‐phase CMRA and conventional multi‐slice 2D CINE images. Additionally, the feasibility of the proposed method was tested in patients with known coronary artery disease. Application of respiratory and cardiac motion correction to both PET and MRI allowed for visualization of the coronary anatomy by CMRA as well as for an improved delineation of the left ventricular myocardium and other cardiac structures by ^18^F‐FDG PET.
